# Characterizing Twitter Discussions About HPV Vaccines Using Topic Modeling and Community Detection

**DOI:** 10.2196/jmir.6045

**Published:** 2016-08-29

**Authors:** Didi Surian, Dat Quoc Nguyen, Georgina Kennedy, Mark Johnson, Enrico Coiera, Adam G Dunn

**Affiliations:** ^1^ Centre for Health Informatics Australian Institute of Health Innovation Macquarie University North Ryde, New South Wales Australia; ^2^ Department of Computing Faculty of Science and Engineering Macquarie University North Ryde, New South Wales Australia

**Keywords:** topic modelling, graph algorithms analysis, social media, public health surveillance

## Abstract

**Background:**

In public health surveillance, measuring how information enters and spreads through online communities may help us understand geographical variation in decision making associated with poor health outcomes.

**Objective:**

Our aim was to evaluate the use of community structure and topic modeling methods as a process for characterizing the clustering of opinions about human papillomavirus (HPV) vaccines on Twitter.

**Methods:**

The study examined Twitter posts (tweets) collected between October 2013 and October 2015 about HPV vaccines. We tested Latent Dirichlet Allocation and Dirichlet Multinomial Mixture (DMM) models for inferring topics associated with tweets, and community agglomeration (Louvain) and the encoding of random walks (Infomap) methods to detect community structure of the users from their social connections. We examined the alignment between community structure and topics using several common clustering alignment measures and introduced a statistical measure of alignment based on the concentration of specific topics within a small number of communities. Visualizations of the topics and the alignment between topics and communities are presented to support the interpretation of the results in context of public health communication and identification of communities at risk of rejecting the safety and efficacy of HPV vaccines.

**Results:**

We analyzed 285,417 Twitter posts (tweets) about HPV vaccines from 101,519 users connected by 4,387,524 social connections. Examining the alignment between the community structure and the topics of tweets, the results indicated that the Louvain community detection algorithm together with DMM produced consistently higher alignment values and that alignments were generally higher when the number of topics was lower. After applying the Louvain method and DMM with 30 topics and grouping semantically similar topics in a hierarchy, we characterized 163,148 (57.16%) tweets as evidence and advocacy, and 6244 (2.19%) tweets describing personal experiences. Among the 4548 users who posted experiential tweets, 3449 users (75.84%) were found in communities where the majority of tweets were about evidence and advocacy.

**Conclusions:**

The use of community detection in concert with topic modeling appears to be a useful way to characterize Twitter communities for the purpose of opinion surveillance in public health applications. Our approach may help identify online communities at risk of being influenced by negative opinions about public health interventions such as HPV vaccines.

## Introduction

The human papillomavirus (HPV) vaccine was first introduced to reduce the incidence of HPV and the majority of cervical cancers [1]. Despite evidence of its safety and efficacy [2-5], the quality of information about the vaccine on the Web is varied [6,7], and coverage of the vaccine is low in some countries, including the United States [8]. For vaccines generally, there is evidence to suggest that negative information about vaccines from celebrities, health practitioners, and news media can increase vaccine hesitancy and refusal [9-11]. Although the HPV vaccine is still a recent addition to the armament of public health, it is important to perform surveillance on social media to understand various opinions about vaccination.

The use of social media information in public health applications has previously centered on forecasting clinical outcomes that have traditionally been measured using surveys and registries. Applications of data mining using Twitter have included influenza surveillance [12-14] and measuring spatial differences in language or mood [15,16]. Sentiment and language analyses on Twitter have been used as indicators for the geographical variation in heart disease mortality [17]. Examples that are relevant to our research include the use of topic modeling to extract tobacco-related tweets in the United States [18] and the surveillance of information about the misunderstanding and misuse of antibiotics from online media [19]. There is a growing area of research considering the spread of information, news, and opinions about vaccines [20-24], and research in this area focuses on measuring associations between misinformation, beliefs, and decision making across a range of community health practices [25].

People interact and form relationships to each other on social media. With these relationships, communities are formed. The structure of online communities influences—and can be influenced by—the information that enters and is diffused through them. Studies examining the spread of information through online communities, social media, and news media have shown that the heterogeneous social structure of the network and external factors can play a role in how far and fast new information spreads [26,27]. That competition between memes and natural attrition may affect how far and fast they can spread and how quickly they decay [28-30], and that topics of interest in a community can also influence the formation and persistence of social structure [31].

Differences in the content of the information posted in online news and social media influence the variation in opinions and beliefs in online communities. While the opinions held by community members and the content they post are not equivalent, we assume that the content provides a reasonable proxy for the opinions that might influence decision making. Topic modeling methods are appropriate for identifying thematic structure (topic) within a set of tweets because they can be applied to an unstructured corpus of documents and there is no requirement that the topic be defined in advance [32]. The primary challenge associated with applying topic modeling to tweets comes from the short length of tweets (140 characters). Despite this challenge, topic modeling has been used to examine topics across a range of subjects on Twitter—by pooling tweets to produce longer documents to analyze [33-35], or applying extensions or alternatives to existing models that work better on shorter documents [36-40].

We expected to find that homophily and contagion would lead to a clustering of opinions in online communities, but to date there has been little research done to measure this important information for vaccines. Our aim was to evaluate the combination of community structure and topic modeling methods in measuring the distribution of topics about HPV vaccines from the tweets posted by users within communities on Twitter, with the broader goal of evaluating a new process for characterizing online communities by the public health information expressed by the community members.

## Methods

### Study Data

Using repeated searches via the Twitter Search Application Programming Interface, we collected tweets about HPV vaccines between October 1, 2013, and October 29, 2015, using the keywords shown in Table 1. For each tweet labeled as English language by Twitter, we stored the text of the tweet and the related metadata. Each time a new user tweeted about HPV vaccines for the first time in the period, we additionally collected the lists of users they followed and who followed them. This information on relationships was used later to construct the network of users for our analysis. At the conclusion of the data collection period, there were 302,856 tweets (including retweets) and 112,944 users. Following the data collection, we removed users who were suspended, protected, or deleted, which left 101,519 users and 285,417 tweets for the analysis.

**Table 1:** Search keywords used to collect tweets about HPV vaccines for our analysis.

No.	Keywords
1.	“HPV” and “vaccine”
2.	“HPV” and “vaccination”
3.	“gardasil”
4.	“cervical” and “vaccination”
5.	“cervical” and “vaccine”
6.	“cervarix”

We pre-processed the tweets prior to topic modeling. For words that were hashtags (beginning with “#”) or usernames (beginning with “@”), we made no further modifications. The remaining words in the text of the tweet were converted to lowercase, and we removed stop words, the word “RT” (which represents a retweet), and any numerical values. We then applied the Porter stemmer [41]. We excluded URLs (uniform resource locator) generated from generic URL shortening services (eg, “http://bit.ly”) and included the domain of any full URLs identified from the list of expanded URLs. Document size plays an important role in topic modeling methods [42], so we chose to assign all of the tweets with fewer than three words to a single extra topic (1114 tweets), leaving 284,303 tweets.

We ran Latent Dirichlet Allocation (LDA) and the Dirichlet Mixture Model (DMM) to infer the topics of the 125,003 unique tweets that were identified within the set of 284,303 tweets after pre-processing the text. After inferring the topics using LDA and DMM, we mapped those topics back onto the full set, so that each tweet was associated with a single topic.

We constructed the network from the 4,387,524 follower connections among the 101,519 users using an undirected graph. A node represents a user, and an edge between two users is established if one was found to be following the other. The network included 100,826 (99.32%) users comprising the single largest connected component, 500 (0.49%) users who formed smaller islands disconnected from the largest connected component, and 193 (0.19%) disconnected users with no connections to the core. Within the largest connected component, the average number of social connections was 86.98 and the largest number of connections was 18,635. We measured the alignment between topics and communities for the users who were part of the largest connected component. More details on network construction are provided in Multimedia Appendix 1.

### Community Detection

Community detection algorithms aim to find sets of nodes in a graph that have a greater density of connections within their set compared to across sets. Traditionally, community detection algorithms produced a hard clustering—where each node belongs to only one community [43-47]. Some of the more recent methods have considered overlapping communities [48]. In this work, we chose two algorithms that assign each node to a single community, are known to produce reliable results, and work efficiently in large networks.

The Infomap algorithm was developed to extract community structure in large complex networks [49]. Using random walks as a proxy for the way that information flows through a system, the method first determines the probability of visiting each node in the network and then characterizes the community and node structure of the network as a Huffman code. By progressively modifying the community affiliation, the aim is to compress the code describing the network to its smallest size. We used the implementation of Infomap from igraph [50].

The Louvain algorithm is a relatively fast community detection algorithm to compute and can therefore scale to large networks [51]. The algorithm is agglomerative—nodes are initialized to belong to a community of size one and sequentially aggregated with the neighboring community that produces the greatest gain in modularity (if a positive gain exists). Communities detected in this first phase become nodes in a new network with edge weights determined by the number of connections between the communities from the first phase. The algorithm therefore constructs a hierarchical representation of the network and proceeds until no more modularity gains can be identified. The final clustering that results from this procedure is used to define the community structure. We used the code released by the author of Louvain from MapEquation [52].

### Topic Inference

Topic modeling is used to find natural clusters based on the co-occurrence of words. We used the Latent Dirichlet Allocation (LDA) model [53] and the Dirichlet Multinomial Mixture (DMM) model [54]. The LDA model is a standard method for topic modeling, and the DMM model is a variant especially developed for short documents such as tweets. When applying the DMM model, only one topic is assigned to each document, so we labeled each tweet according to the topic inferred by the DMM model. For LDA—where a probability topic distribution is produced for each document—tweets were labeled using the topic with the largest probability [33,40,55]. We used the implementation of LDA from gensim [56] and the jLDADMM implementation of DMM [57]. For both methods, we used standard settings for each model [39,55] and did not attempt to optimize the parameters further. Details of the formal specification and notations for the methods are provided in Multimedia Appendix 1.

### Alignment Measures

The aim of measuring the alignment between topics and communities is to determine if the topics appear more frequently within some communities relative to all others. Since each tweet was associated with a single topic, we represented communities by the distribution of topics in the tweets posted by the users in that community. We adapted measures of alignment that are typically used to quantify the quality of an estimated clustering against an observed clustering to compare between the clustering methods that use the observed structure (social connections) and the clustering methods that use the observed content (topics in tweets). There are several appropriate metrics for assessing cluster quality in this scenario, including purity, normalized mutual information (NMI), and the adjusted Rand index (ARI) [39,58] (see Multimedia Appendix 1 for definitions).

While these typical metrics provide a general measure of the alignment between community structure and the topics of the tweets posted by users in those communities, they were not useful for summarizing how topics may be disproportionately represented within a small subset of the communities. We therefore additionally considered a measure of topic concentration (*TC*). We defined a *TC* value by the smallest number of communities required to cover a specified percentage of the tweets about a given topic, so *TC*_95_ is the number of communities required to cover 95% of the tweets in that topic, and *TC*_100_ is the number of communities that covers every tweet labeled with that topic. A lower *TC*_95_ value therefore implies a higher concentration of topics within a small number of communities.

When comparing measures of topic concentration across multiple networks to determine alignment, the differences in the number of tweets associated with each topic can influence the measures independently of the alignment, so we used permutation tests to produce a fair comparison. The permutation tests create a baseline distribution of *TC* values that may occur in the absence of any real alignment, which can then be used to establish the level of alignment relative to the levels of alignment that could be produced by chance [59]. To do this, we randomly permuted the topics associated with each tweet such that the distributions of tweets per topic and tweets per community remained the same as the observed network. We then compared the observed *TC*_95_ values against the distribution of *TC*_95_ values produced in the permutation tests. Typical permutation tests report the percentile of a single observed value within the distribution of values produced after permutation. In the permutation tests we applied, distributions of *TC*_95_ values (one for each topic) were produced rather than single values, so we used a two-sample Kolmogorov Smirnov test to compare the distributions. The Kolmogorov-Smirnov test statistic varies between 0 and 1, and a higher test statistic means that the topics were more concentrated within individual communities than would be expected if the same number of tweets per topic were randomly distributed across the communities.

### Manual Intrusion Tests

We performed intrusion tests on the topics from the tweets. One investigator, blinded to the results of the topic modeling, was presented with sets of five test cases per pairwise combination of topics. Each test case included the text of five tweets chosen at random from one topic and one tweet chosen at random from a different topic. The investigator was tasked with identifying the tweet that did not belong to the topic. The results of these intrusion tests indicated how well the topic modeling was able to capture semantic differences in the tweets. We additionally used the results of the intrusion tests to construct a hierarchy of topics based on their semantic dissimilarity by applying multidimensional scaling [60-62]. The method produces a distance between every pair of topics, which is then used to merge the closest topics to construct the hierarchy.

## Results

### Community Detection and Topic Modeling

The two community detection algorithms were applied to the largest connected component of 100,826 users. Applying the Louvain algorithm, we identified 38 distinct communities of sizes between 3 and 21,733 users. The Infomap algorithm identified 1334 distinct communities, ranging in size from 2 to 18,974 users.

We constructed a series of LDA and DMM models by varying the number of topics between 5 and 200. From the purity, NMI, and ARI scores, we found that the alignment between the community structure and the topics was higher across all measures for DMM compared to LDA. The highest purity score (0.495) and the highest ARI scores (0.166) were found when applying the DMM model with the Louvain algorithm. The highest NMI score (0.185) was found when applying the DMM model with the Infomap algorithm. The results of these experiments suggest that the DMM topic model may have produced a more realistic clustering of the tweets by topic.

The *TC*_95_ scores were consistently higher when using the DMM model compared to the LDA model (see Multimedia Appendix 1 for detailed results). In combination with the Infomap algorithm, *TC*_95_ scores were highest between 10 and 25 topics, and in combination with the Louvain algorithm, *TC*_95_ scores were highest between 20 to 30 topics. Considering these results, we used the DMM model (with 30 topics) and the Louvain algorithm to demonstrate the characterization of the communities by topic in what follows.

**Figure 1:**
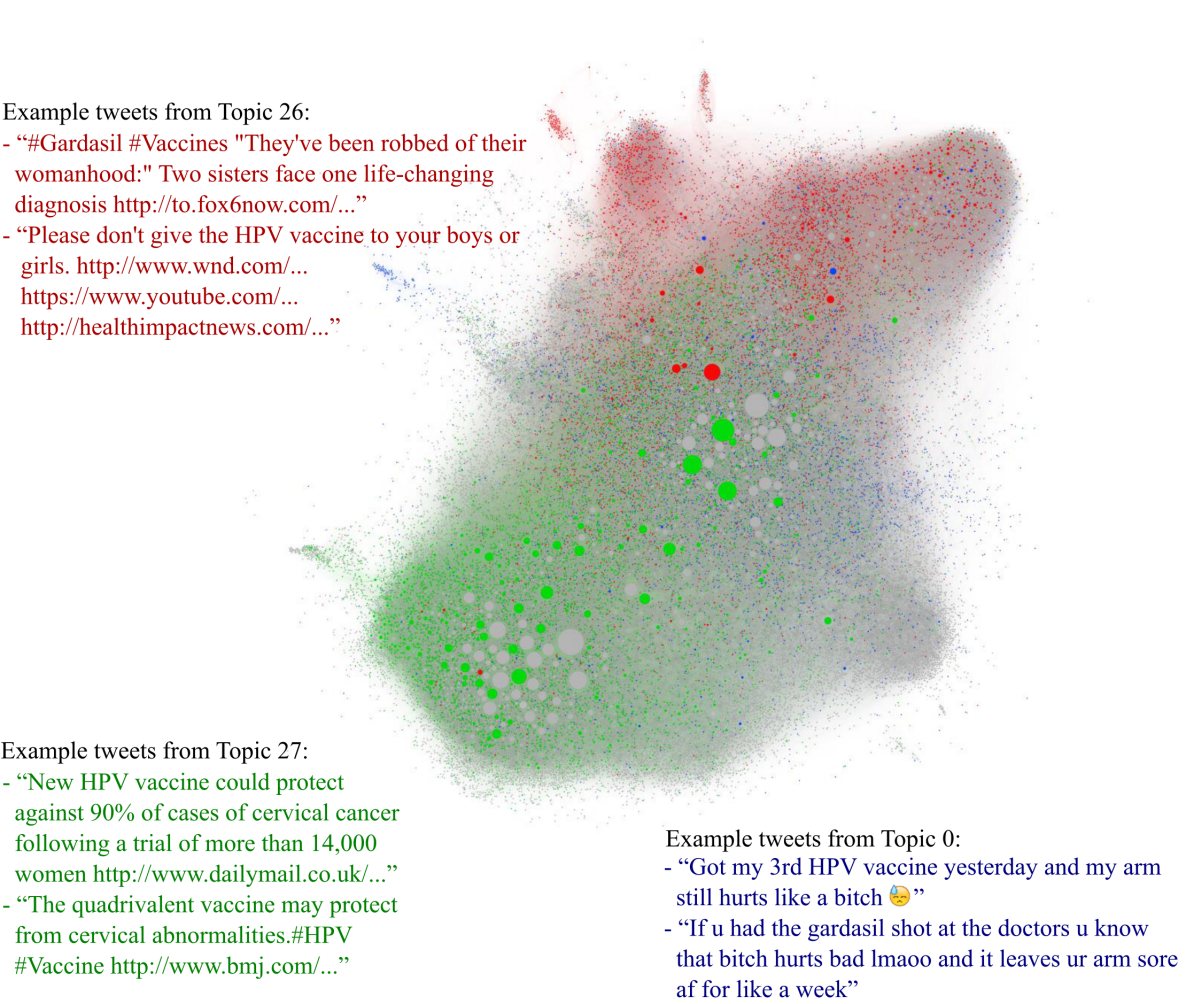
A network of 100,826 users (nodes) who posted tweets about HPV vaccines in the period. The sizes of the nodes are proportional to the number of social connections they have in this network. Nodes are colored if they posted tweets labeled as Topic 0 (blue), Topic 26 (red), or Topic 27 (green). Node position was determined by a heuristic that attempts to locate connected nodes closer together, partially revealing the community structure.

To illustrate how the topics tend to cluster within communities, we selected three representative topics and visualized them in the network constructed from the set of followers among the 100,826 users (Figure 1). The topics include one of the topics that captured clinical and scientific evidence (Topic 27), the topic comprising experiential tweets (Topic 0), and one of the topics describing side effects and harms (Topic 26).

Topic 27 includes words that are common to published studies about the efficacy of the vaccine such as “prevent,” “protect,” “study,” “news,” and “research.” Links to news media alongside other published articles and related media tended to be grouped within this topic, and the topic is broadly represented throughout the majority of the core network, including among the users with the greatest number of connections (typically news organizations in the center, and news organizations, health-related magazines, and scientific journals to one side).

Topic 0 captures a large number of tweets from users describing their own experiences with the vaccine, including temporal words such as “today,” “get,” “got,” and “go.” Tweets including phrases such as “my arm hurts like a...” were commonly assigned to this topic, and these users appeared to share fewer connections with other users posting about HPV vaccines.

In Topic 26, emotive words like “kill,” “victim,” and “death” are common. Tweets that include links to specific antivaccine websites were commonly assigned to this topic, and users posting tweets in Topic 26 appeared to cluster with different densities in three distinct groups that were separated from the groups of users posting tweets labeled as Topic 27.

### Topic Grouping

**Figure 2:**
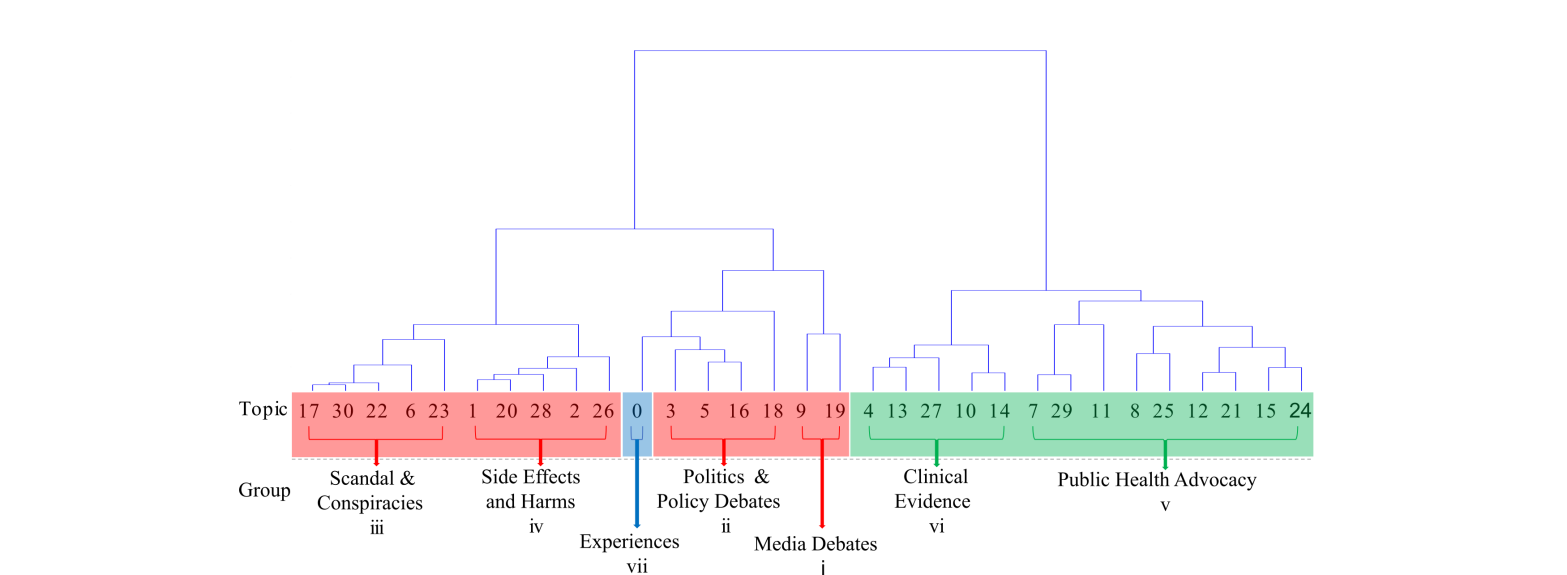
A dendrogram of 30 topics (Topic 0-29) from the Dirichlet Mixture Model and one separate topic (Topic 30) for the tweets with fewer than 3 words. The groups were identified post-hoc and the colors represent themes—harms/conspiracies (red), evidence/advocacy (green), and experiential (blue).

We measured the quality of the topic modeling using the manual intrusion tests. Overall, the correct intruder was identified in 63.7% of the 4650 tests, which is a clear departure from the 16.7% that would be expected by chance. The hierarchy constructed from the manual intrusion tests revealed the semantically similar topics (Figure 2). The topic groups were (1) media debates, (2) politics and policy debates, (3) scandals and conspiracies, (4) side effects and harms, (5) public health advocacy, (6) clinical evidence, and (7) experiences. When measured across the groups of topics, the intrusion test accuracy was 76% and when measured within the groups of topics, the intrusion test accuracy was 49%. These results suggest that the separation among topic groups is clear (high score for intergroup accuracy and low score for intragroup accuracy).

**Figure 3:**
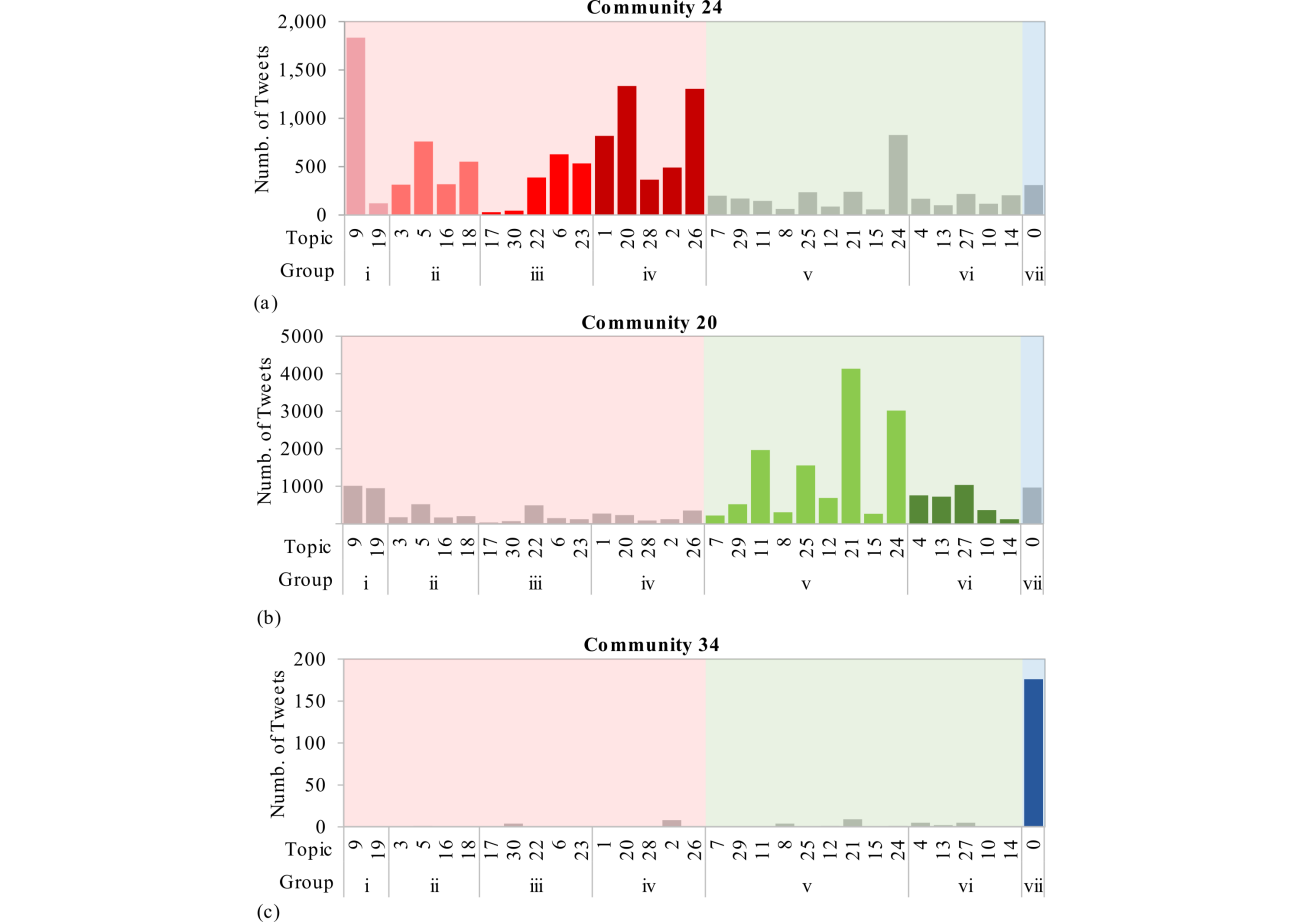
Topic distributions for 3 selected communities ordered by group and theme: (1) community 24 included 5275 users and an average of 2.46 tweets per user; (2) community 20 included 11,047 users and an average of 1.96 tweets per user; and (3) community 34 included 187 users and an average of 1.16 tweets per user.

Using the topics groups, we were then able to characterize the communities by the distribution of topics among the set of tweets posted by the users in those communities. Figure 3 details the topic distributions for three selected communities, notable because they illustrate the concentration of vaccine harms/conspiracies, evidence/advocacy, and experiential themes within different communities. Note also that the number of tweets per user is highest for users in the community that posts tweets labeled mostly among the vaccine harms/conspiracies theme and lowest for users in the community posting mostly about their experiences with the HPV vaccine.

**Figure 4:**
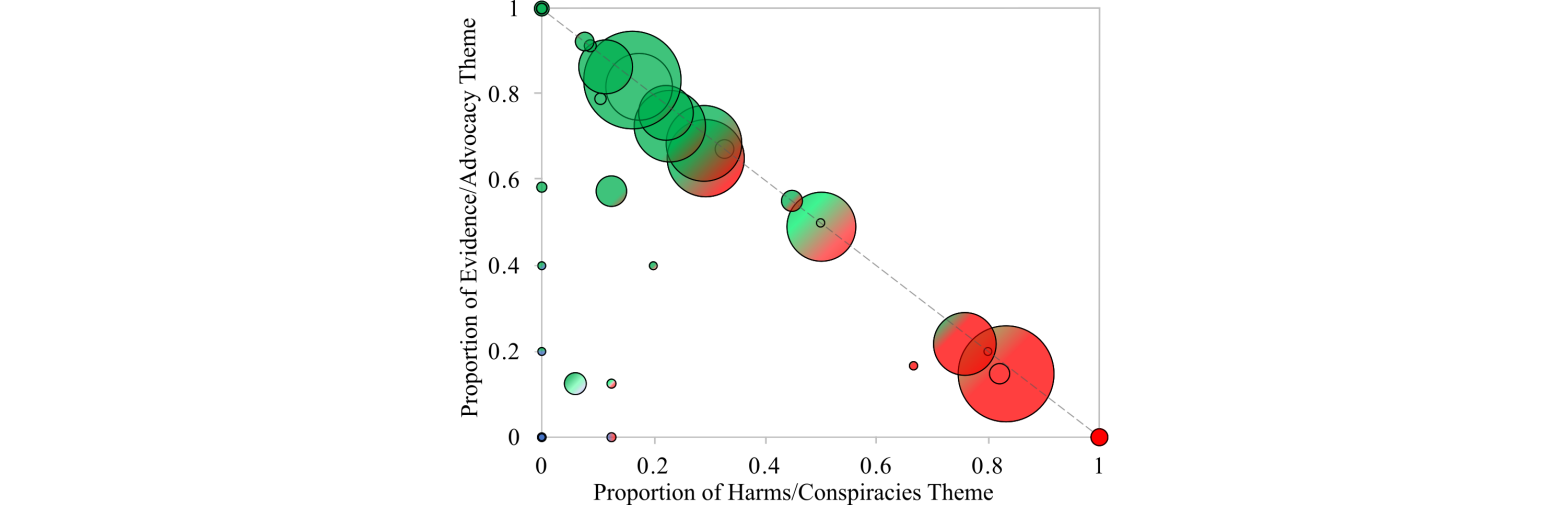
The proportion of evidence/advocacy and harms/conspiracies themes for the identified 39 communities. Each circle represents a community and the size is proportional to the number of tweets in the respective community. Communities further from the diagonal include greater proportions of experiential theme tweets.

**Figure 5:**
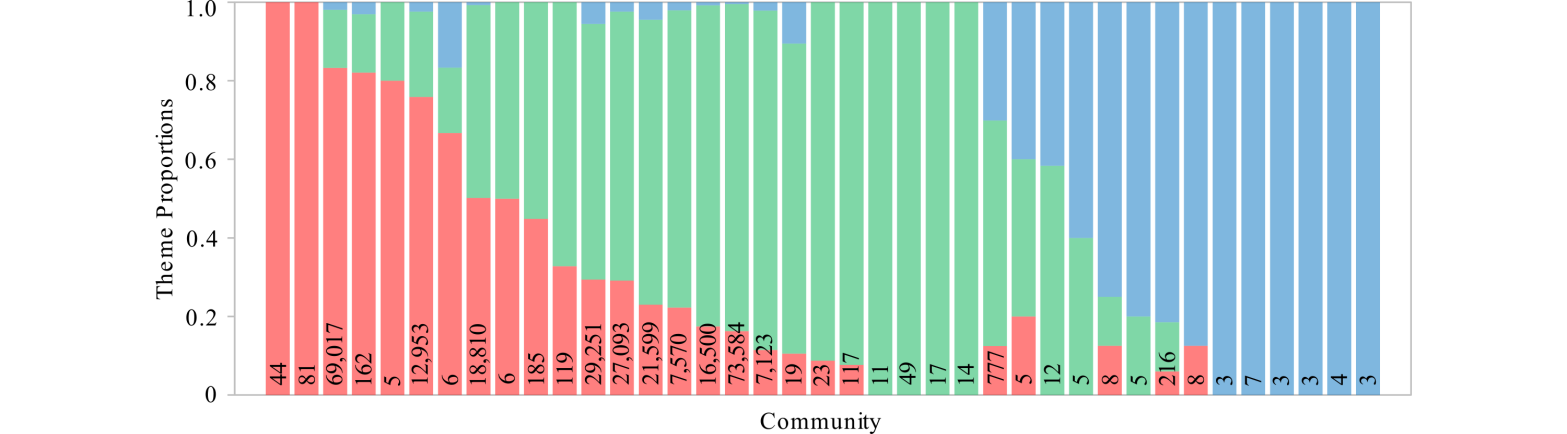
Theme proportions across the 39 communities, representing the proportion of tweets that were assigned to the themes of harms/conspiracies (red), evidence/advocacy (green), and experiential (blue). Values along the horizontal axis are the total number of tweets in the community.

Across the set of all communities, we found that users posting about their own experiences with the HPV vaccine belonged to communities for which the majority of tweets were related to evidence and advocacy. Of the 4548 users who posted tweets labeled as experiential, 3449 (75.84%) belonged to communities for which the majority of tweets were related to evidence/advocacy, 674 (14.8%) belonged to communities for which the majority of tweets were related to harms/conspiracies, 196 (4.3%) belonged to communities for which the majority of tweets were experiential, and 229 (5.0%) belonged to the group of users who were not connected to the core of the network. Figures 4 and 5 detail the distribution of themes within the communities.

## Discussion

### Principal Results

In this study, we sought to measure the alignment between the community structure implicit among the follower network of Twitter users posting tweets about HPV vaccines and the topics about which they posted. Given what is already known about the variable quality of information about HPV vaccines on Twitter [22,23], we expected to find that some communities would more often perpetuate negative opinions about HPV vaccines and that these communities would be distinct from the communities describing the favorable evidence or advocating for its uptake. Using a statistical measure quantifying the strength of the topic concentration, we found that some topics were heavily concentrated within a small number of communities, which was consistent with our expectations. Compared to our previous work in this application domain [22,23], the process described here provides a more nuanced view of the specific concerns about HPV vaccines expressed on social media and the ability to identify communities in which these concerns were the predominant topics. Using the combination of topic modeling and community structure to characterize communities, we were able to identify communities in which specific concerns about safety or politics were predominant, as well as identify younger Twitter users who posted experiential tweets and were at risk of greater exposure to safety concerns than to evidence and advocacy, which may occur between the first and subsequent doses of the vaccine. Analysis of tweets for public health where opinions and experiences are mixed have been investigated previously for influenza, where some tweets may help identify influenza incidence and others represent evidence dissemination or opinion [21].

### Comparison With Prior Work

A growing set of methods has been developed using either structural information to improve inferences about the content of a corpus, or the information characterizing the nodes in a network to improve the analysis of the structure. Those aiming to understand the content of tweets have used social connections to improve tweet classification [22,63-65]. These studies have considered mentions, retweets, and other forms of interaction that are available on Twitter, but the use of information about followers generally produced the highest levels of performance. Other researchers have proposed methods for incorporating network structure into topic modeling approaches in networks other than Twitter [66,67]. Conversely, some studies have considered the use of content associated with nodes in networks to improve the quality of community detection [68,69]. Among the studies examining documents and the structure between them—such as emails [70], co-authorship [71], and Wikipedia [72]—one study produced topic profiles for communities in a similar fashion to the way we have done in Figure 3 [73]. The approach we presented here differs from these studies because we applied community detection and topic modeling independently, rather than attempting to leverage the information available about social connections to improve the quality of the topic modeling process, or to use content information to improve community structure or predict new connections.

### Limitations

A limitation of this work is that we considered a single application domain. While the uptake of vaccines is of critical importance to public health, further testing on other application domains would be required to determine the generalizability of assessing topic concentrations as a way of characterizing Twitter communities. A further limitation in our work is that we did not consider the temporal dynamics of the topics or the community structure in any detail. Given that the topics related to HPV vaccines are likely to produce similar temporal patterns to those observed by Leskovec et al [30], future work in this area may benefit from further analysis of the relationship between the temporal dynamics of the topics and the economy of attention within communities, which has been explored elsewhere [28,29]. Finally, we considered the follower network as an undirected network and did not incorporate weights or directionality based on mutual followers, or the presence of retweets and mentions, which would have provided a more nuanced representation of the social connections and may have produced a different community structure.

### Future Directions

Our work here has potential implications for public health practices. Applying topic modeling and community detection methods in concert to a corpus of tweets about HPV vaccines, we found that it was possible to characterize online communities by the topics that are most heavily concentrated among their tweets. One way of translating these methods into public health practice would be to use these methods in combination with new spatial and demographic estimation methods [74-76], to produce spatiotemporal indicators that determine where and when the growth of specific concerns may lead to increased vaccine hesitancy or refusal. We think that these indicators may have a future role in helping public health organizations design interventions and communication strategies that are better targeted and thus more efficient.

### Conclusions

In this work, we demonstrated a novel process for characterizing the concentration of certain opinions in online communities by independently applying existing community detection and topic modeling methods, and quantifying the differences in the topic distributions across communities. Among tweets about HPV vaccines, we found that there were clear differences in the distribution of topics across communities defined by the follower network. In practice, public health organizations may wish to consider identifying the locations and demographics of communities that are at risk of exposure to antivaccine information in order to intervene with positive messages targeting the specific concerns identified through topic modeling. The value of this work in public health includes a more nuanced representation of the variety of concerns expressed about HPV vaccines online and some practical steps towards the development of an automated system for the surveillance of public opinions with the purpose of understanding localized differences in decision making and health behaviors.

## Appendix

Multimedia Appendix 1 Network construction, formal specification/notations, and detailed experiment results.

## Abbreviations

ARI: adjusted Rand index
DMM: Dirichlet Multinomial Mixture
HPV: human papillomavirus
LDA: Latent Dirichlet Allocation
NMI: normalized mutual information
