# Supplementary material for: Characterizing Twitter Discussions About HPV Vaccines Using Topic Modeling and Community Detection
Source: J Med Internet Res. 2016 Aug 29;18(8):e232. doi: 10.2196/jmir.6045 (PMC5020315; doi:10.2196/jmir.6045)
Supplement: Multimedia Appendix 1 [file jmir_v18i8e232_app1.pdf]

Appendix

Network Construction

We use an undirected graph G to represent the social connections and potential information flow within a cohort of Twitter users. In $G = (V, E)$, V denotes the set of nodes (Twitter users) and E denotes the set of edges (social connections) in G. An edge $e_{ij} \in E$ corresponds to a set of node pairs (v_i, v_j) that connects node v_i and v_j in G. To define an edge in the network, we collected information about the users, including the lists of users they followed (following) and the lists of users following them (followers). Links were established between two users if one was found to be following the other. The goal of non-overlapping community detection in G is therefore to find k subsets (communities) of V, $\{V_0, V_1, \ldots, V_k\}$, $V_i \cap Vj = \emptyset$ for $i \neq j$ and $\cup_i V_i = V$.

Topic Inference

Latent Dirichlet Allocation

Latent Dirichlet Allocation (LDA) is a probabilistic model that learns latent structure (or topics) of a corpus of text documents [1]. LDA is based on "bag-of-words" assumption where the order of words is ignored and follows a theorem by de Finetti [2] which states that any collection of exchangeable random variables can be represented by a mixture of distributions. LDA uses several basic assumptions: a document is represented by a mixture of a finite number of K topics and a topic is represented by a distribution over words. Figure A1 shows the graphical model of LDA.

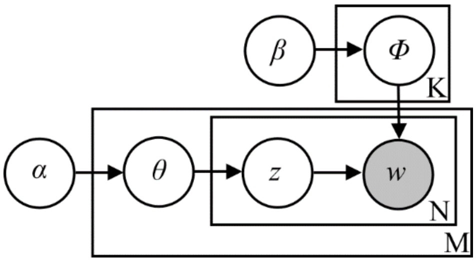

Figure A1. Graphical model of LDA

The plate (rectangle) represents a repetition of a variable (e.g. words in a document) and a circle represents the variable. The observed variable (e.g. words, *w*) is represented by a shaded circle, while an unobserved variable is represented by an unshaded circle (e.g. topic mixture of a document, θ, topic assignment of a word, *z*, and distributions over words for topics ϕ).

Assume that there are M documents, each document $m \in \{1,\ldots,M\}$ has N_m words and we specify that there are *K* topics. LDA is based on the following generative model of words in documents. For each topic $k \in \{1,\ldots,K\}$, LDA generates a distribution over words from the Dirichlet distribution with a hyper-parameter β, $\phi_k \sim Dir(\beta)$. The topic mixture for each document *m*, θ_m, is generated from the Dirichlet distribution with a hyper-parameter α, $\theta_m \sim Dir(\alpha)$. To generate word w_{mn}, LDA first chooses a topic assignment z_{mn} from the multinomial distribution θ_m, $z_{mn} \sim Mult(\theta_m)$. Finally, a word is generated from the multinomial distribution conditioned on topic z_{mn}, $w_{mn} \sim Mult(\phi_{k=z_{mn}})$. The generative process of LDA is summarised as follows:

1. For each topic $k \in \{1,\ldots,K\}$, generates a distribution over words from the Dirichlet distribution with a hyper-parameter β, $\phi_k \sim Dir(\beta)$.
2. For each document $m \in \{1,\ldots,M\}$, sample topic proportions θ_m using the Dirichlet distribution with symmetric hyper-parameter α: $\theta_m \sim Dir(\alpha)$, where $\Sigma\theta_m = 1$ and the dimension of θ_m is *K*.
3. For each word $w_{mn} \in \{1,\ldots, N_m\}$ in document *m*:
   a. Choose a topic $z_{mn} = k$, $k \in \{1,\ldots,K\}$ from θ_m using the multinomial distribution: $z_{mn} \sim Mult(\theta_m)$
   b. Choose a word w_{mn} from a multinomial probability conditioned on the topic assignment z_{mn}, $p(w_{mn}|\ z_{mn}, \beta)$: $w_{mn} \sim Mult(\phi_{k=z_{mn}})$.

The goal of topic model is to learn the latent variables which is a Bayesian inference problem. Gibbs sampling [3], variational Bayes [1] and expectation propagation [4] are commonly used to solve the inference problems.

Dirichlet Mixture Model

The Dirichlet Multinomial Mixture (DMM) model is a generative model that differs from LDA in that each document m is associated with a single topic z_m rather than a distribution over topics as in LDA [5]. Thus, DMM is a mixture model, whereas LDA is an admixture model. Recently, Yin et al. [6] showed the DMM achieved significantly better performance for short text clustering tasks such as on Twitter data set. Figure A2 shows the graphical model of DMM.

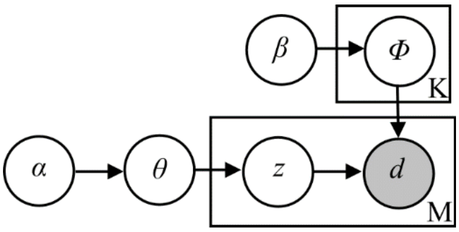

Figure A2. Graphical model of DMM

In DMM, the observed variable is the bag of words in a document, just as in LDA. The generative process of DMM is described as follows. For each cluster (topic) $k \in \{1,\ldots,K\}$ DMM generates a distribution over words from the Dirichlet distribution with a hyper-parameter β, $\phi_k \sim Dir(\beta)$. The mixture weights (topic mixture) θ are generated from the Dirichlet distribution with a hyper-parameter α, $\theta \sim Dir(\alpha)$. To generate a document $d_m \in \{1,\ldots,M\}$ DMM first chooses a cluster label (topic) z_m from the multinomial distribution on θ, $z_m \sim Mult(\theta)$. The DMM then generates the words in document d from the multinomial distribution conditioned on cluster label (topic) z_m, $w_m \sim Mult(\phi_{k=z_n})$. In brief, the probability of document d generated by cluster (topic) k is $p(d|z=k)=\Pi_{w \in d}\, p(w|z=k)$.

Alignment Measures Results

Cluster alignment

The adjusted Rand index (ARI) is an extended version of Rand index (RI), which measures the percentage of tweets with the same topics being grouped into same community and tweets with different topics into different communities. An ARI assumes the generalised hypergeometric distribution as the model of randomness. Thus an ARI score is bounded above by 1 and close to 0 is expected if tweets are distributed at random among the communities. The ARI is defined as:

$$\mathrm{ARI}=\frac{\mathrm{RI}-\mathrm{E}[\mathrm{RI}]}{\max(\mathrm{RI})-\mathrm{E}[\mathrm{RI}]},\ \mathrm{RI}=\frac{\mathrm{P+Q}}{Comb_2^N}$$

where *P* (*Q*) is the number of pairs of elements that are in the same (different) set in *R* and *K*, and $\mathrm{Comb}_2^{\mathrm{N}}$ is the total number of possible pair combinations.

The normalised mutual information (NMI) measure determines how similar the joint distribution of two random variables is to the products of their factored marginal distributions and is defined as follows:

$$\mathrm{NMI}=\frac{I(A,B)}{\sqrt{H(A)H(B)}}$$

where *H* represents marginal entropy, *I* represents mutual information, $A=\{a_1,...,a_N\}$ represents community labels, and $B=\{b_1,\dots,b_N\}$ represents topic assignments. A value close to 0 represents poor alignment, while a value of 1 represents perfect alignment between the community structure and the topics.

The purity of a community is the number of elements of the largest class (topic assignment) in the community divided by the total number of tweets in the community. Thus, the purity is defined as:

$$\mathrm{purity}=\frac{1}{N}\sum_{r=1}^{R} argmax_k(n_r^k)$$

where n_r is the size of particular community V_r, n_r^k is the number of tweets in the community V_r that are assigned to topic *k*. A purity close to 0 indicates a poor alignment between the community structure and the topics, and a purity of 1 represents a perfect alignment.

The ARI, NMI and purity were used in an attempt to quantify how often individual topics were concentrated within a small number of communities. To do this, we compared clusters of tweets by topic with clusters of tweets by community—defining a community cluster by the set of tweets posted by any users within that community, and a topic cluster as all the tweets that were assigned to that topic. Figure A3 shows the ARI, NMI and purity scores for combinations of DMM and LDA with Louvain and Infomap for number of topics 5 to 200.

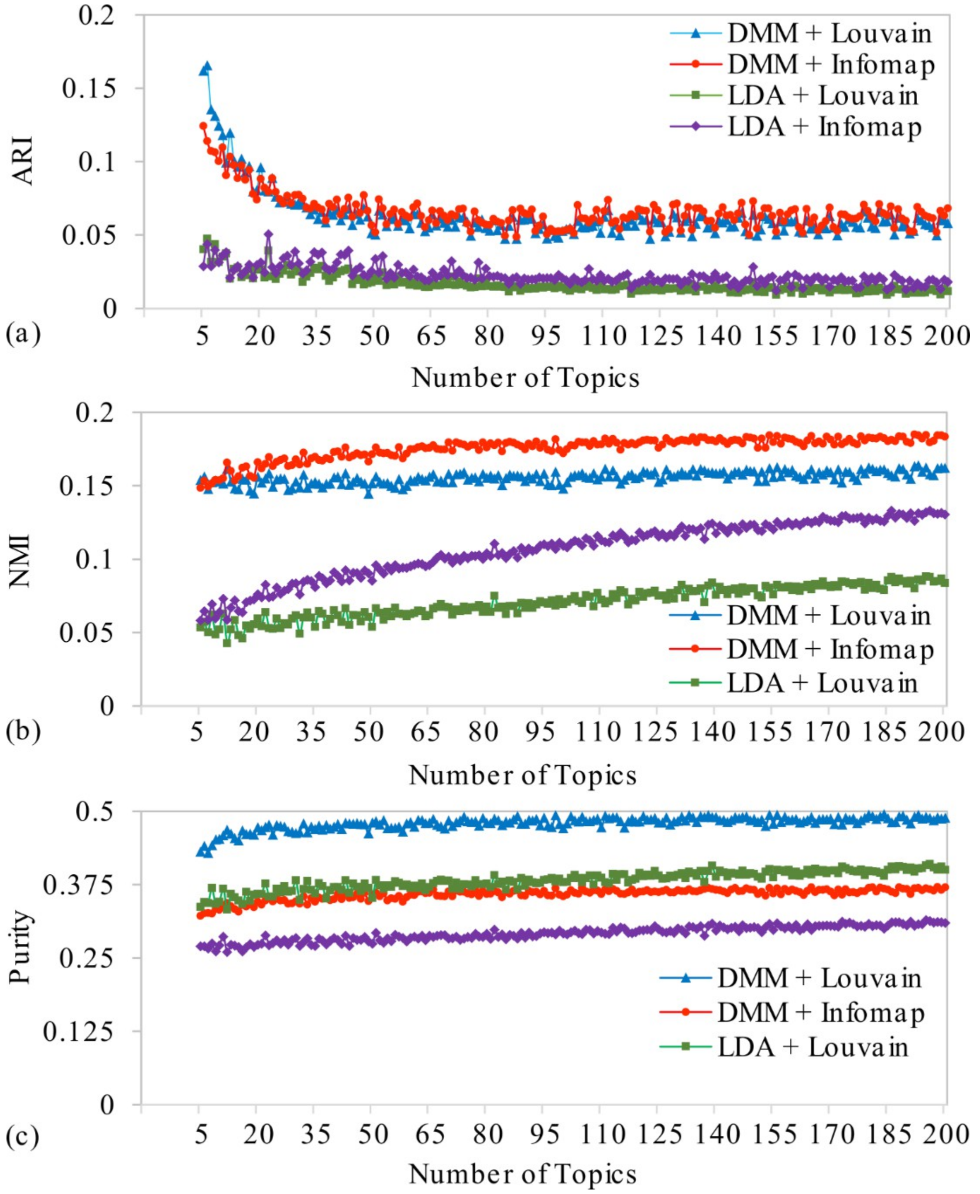

Figure A3. The (a) ARI (b) NMI and (c) Purity scores for number of topics 5 to 200 for Latent Dirichlet Allocation (LDA) and Dirichlet Mixture Model (DMM) on Louvain and Infomap. Note that the vertical axes in the subfigures have different scales. Larger scores (left vertical axis) close to 1 mean good alignment between the community structure and the topics.

In general, the alignment between the community structure and the topics was higher across all measures for the DMM method compared to the LDA method. Under the assumption that we expected to observe a concentration of some topics within a small number of communities, and given that the topic modelling was undertaken without any consideration of the social connections between users, the results of these experiments suggest that the DMM method may have produced a more realistic clustering of the tweets by topic.

Individual topic concentration

We found that in the combination of the DMM method for topic modelling (with the number of topics set to 30) and the Louvain method for community detection, a random assignment of the 30 topics across the set of tweets without any consideration of the structure most often required 9 communities to cover 95% of any topic. In the observed topic distribution, the TC_{95} values range between 6 and 11 communities, and the majority of topics are 95% covered by 8 or fewer communities. The difference between the two distributions suggests that in the observed network, topics are more concentrated within communities than would be expected by chance.

We calculated the TC_{95} values for all topics with at least one tweet for each combination of the community detection and topic modelling methods, and varying the number of topics between 5 and 200 (Figure A4). We found that when the number of topics was relatively low, the DMM method tended to find topics that had higher levels of concentration within communities.

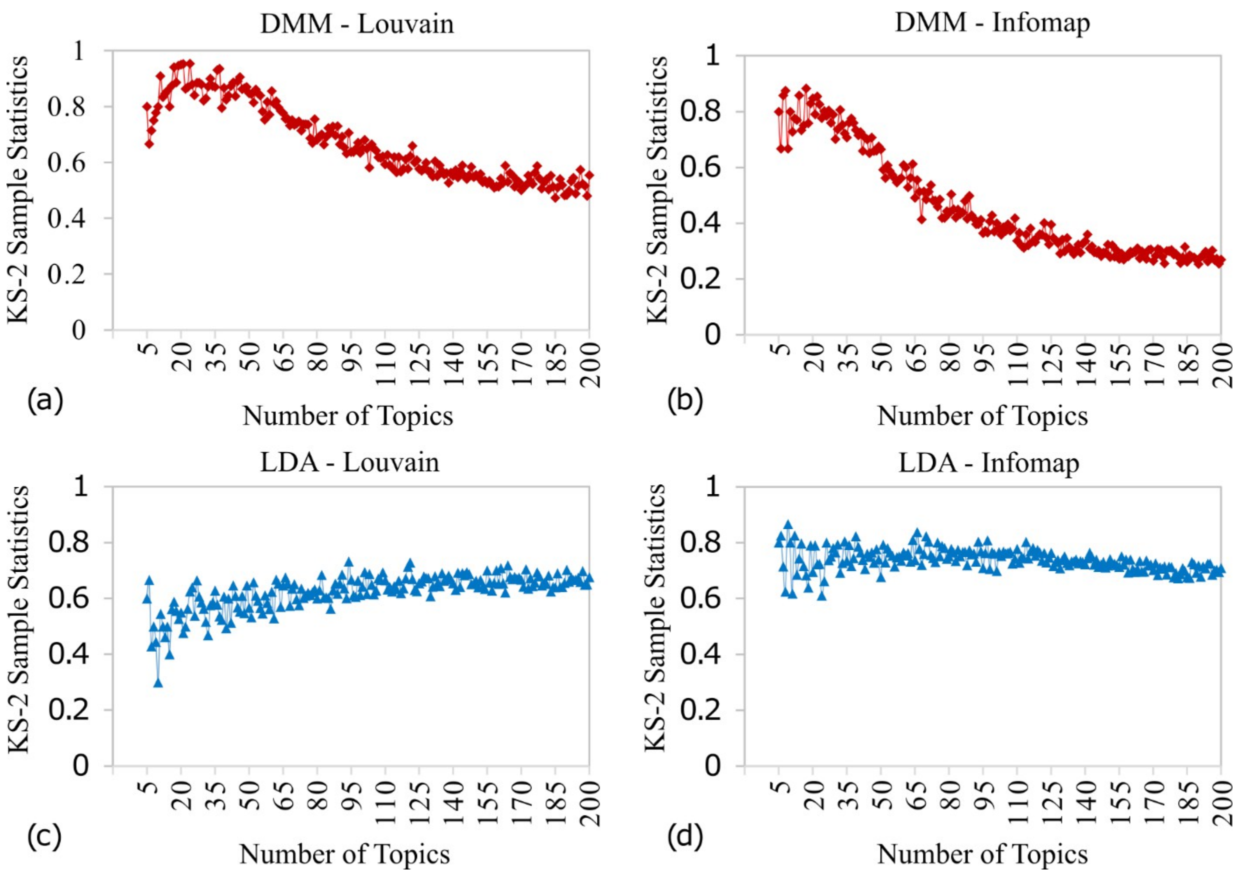

Figure A4. The two-sample Kolmogorov-Smirnov test statistic of (a) DMM + Louvain (b) DMM + Infomap (c) LDA + Louvain and (d) LDA + Infomap comparing the distribution of observed TC_{95} values against the distribution of TC_{95} values from the permutation tests for number of topics from 5 to 200. Higher values on vertical axis represent stronger topic concentration.

Manual Intrusion Test

Figure A5 shows the results of manual intrusion test. Each value represents how many times in five test case the investigator correctly identified the intrusion topics. Higher values mean that the intrusion topics were easily identified.

Intrusion Topics \ Baseline Topics	9	19	3	5	16	18	17	30	22	6	23	1	20	28	2	26	7	29	11	8	25	12	21	15	24	4	13	27	10	14	0
9	0	5	2	3	3	5	4	4	4	5	4	4	4	5	4	4	4	4	4	5	3	4	5	3	2	4	4	3	2	2	5
19	2	0	3	3	4	5	2	2	4	5	5	3	5	5	4	5	1	4	4	3	5	4	5	2	4	3	3	4	1	2	3
3	5	5	0	1	2	5	0	1	0	1	3	3	2	1	4	3	4	5	4	5	5	5	5	2	3	3	5	3	1	2	3
5	4	4	2	0	2	3	2	3	3	4	4	2	5	3	5	3	4	3	5	4	5	4	4	4	2	5	5	5	2	1	1
16	3	5	2	1	0	5	0	3	2	4	5	5	3	3	3	5	5	5	5	4	5	5	5	3	3	5	3	5	1	2	5
18	2	5	3	0	2	0	3	3	3	5	3	4	3	2	5	2	4	5	5	2	3	3	4	4	0	5	5	5	1	3	3
17	4	5	3	2	3	4	0	1	1	1	5	3	4	3	2	3	3	5	5	3	5	5	5	3	2	5	5	3	2	4	3
30	4	5	2	1	2	5	0	0	0	3	3	2	2	3	2	4	1	5	5	3	5	4	5	2	0	5	3	4	2	2	0
22	3	4	1	2	2	5	0	1	0	1	2	0	2	3	3	3	3	5	5	5	5	3	4	2	3	5	4	3	1	2	3
6	5	5	3	1	3	4	0	0	0	0	1	1	3	1	2	0	4	4	5	4	5	5	5	3	3	5	4	5	1	2	2
23	5	5	4	3	2	4	0	2	0	0	0	1	3	0	2	5	4	5	5	5	5	5	5	5	2	4	5	5	3	3	4
1	4	5	1	0	0	4	0	2	0	0	3	0	0	0	4	2	4	5	5	5	5	5	5	3	0	5	3	5	1	3	2
20	4	5	0	1	2	4	0	2	1	1	4	0	0	1	1	0	5	5	5	5	4	5	5	5	3	5	4	5	2	3	3
28	4	5	0	0	1	5	1	2	0	1	2	4	0	0	1	2	5	5	3	5	4	4	5	2	1	5	3	5	1	2	3
2	4	5	3	0	1	5	0	1	0	1	2	0	0	1	0	1	5	5	5	4	5	5	5	5	4	4	5	5	0	2	2
26	4	5	1	0	2	4	0	1	0	0	3	0	1	0	1	0	3	5	5	5	5	5	5	2	0	5	4	5	2	2	4
7	3	5	4	2	4	4	5	4	4	2	5	5	5	3	4	4	0	1	3	2	5	4	2	2	1	1	3	1	1	1	3
29	4	5	5	3	4	5	4	5	5	4	5	5	5	4	5	5	0	0	3	0	3	2	3	0	1	3	1	3	1	3	3
11	5	5	3	2	4	5	3	4	4	5	5	4	5	4	3	5	1	4	0	3	5	4	4	1	1	3	4	5	1	2	3
8	4	4	5	3	5	4	2	1	3	4	5	5	5	5	5	5	1	4	4	0	2	2	2	2	1	2	2	4	3	1	0
25	5	5	3	5	5	4	3	1	3	5	5	4	5	5	4	5	1	4	3	0	0	1	2	2	0	5	2	4	1	2	3
12	3	5	3	0	3	4	3	4	4	5	5	4	4	5	4	5	2	5	3	2	0	0	1	0	1	3	1	3	2	2	1
21	5	5	4	3	4	5	4	3	3	5	4	5	5	5	4	5	0	3	2	3	4	2	0	1	0	1	1	2	2	0	3
15	5	5	4	1	5	4	1	3	3	4	5	5	5	5	4	4	2	4	5	4	2	2	2	0	1	2	3	3	0	1	2
24	5	5	3	3	2	4	2	4	4	4	4	3	5	4	5	4	3	4	4	4	3	3	3	1	0	3	4	5	3	2	2
4	4	5	4	4	2	5	4	3	5	5	5	4	5	4	5	5	4	5	2	2	4	3	1	1	1	0	1	1	2	0	3
13	4	5	3	3	3	4	4	3	4	5	5	5	4	5	3	4	2	5	4	1	3	3	1	0	0	2	0	3	1	1	4
27	5	3	3	4	4	5	3	3	5	5	5	4	4	4	4	5	3	4	5	2	3	3	2	0	1	1	1	0	0	0	2
10	4	4	4	2	3	5	1	3	2	4	5	3	5	2	3	4	3	4	5	1	4	4	3	4	1	3	3	4	0	4	5
14	5	5	5	5	3	5	1	2	3	5	4	4	4	2	2	1	3	5	4	2	5	4	3	2	3	4	3	1	0	0	3
0	5	5	1	2	1	4	3	3	4	4	4	5	3	4	5	4	3	5	5	2	5	4	5	5	1	4	5	3	2	3	0

Figure A5. Manual intrusion test results. The numbers in the table represent how many times the investigator correctly identified the intrusion topics in the baseline topics. The colour on the horizontal and vertical axes represents the themes: harms/conspiracies (red), evidence/advocacy (green), and experiential (blue) themes.

References

1 Blei DM, Ng AY, Jordan MI. Latent Dirichlet Allocation. J Mach Learn Res 2003;3(4-5):993–1022. doi: 10.1162/jmlr.2003.3.4-5.993

2 Finetti BD. Theory of probability: John Wiley & Sons; 1990. ISBN:0-471-92612-4/v.2

3 Griffiths TL, Steyvers M. Finding scientific topics. Proceedings of the National Academy of Sciences (Suppl 1). 2004;101:5228-35. PMID: 14872004

4 Minka T, Lafferty J. Expectation-propagation for the generative aspect model. Proceedings of the 18th Conference on Uncertainty in Artificial Intelligence; 2002 Aug 1-4; Alberta, Canada. San Francisco, CA: Morgan Kaufmann; 2002. p. 352-359

5 Nigam K, McCallum AK, Thrun S, Mitchell T. Text classification from labeled and unlabeled documents using EM. Mach Learn 2000;39(2-3):103–134. doi: 10.1023/A:1007692713085

6 Yin J, Wang J. A Dirichlet Multinomial Mixture model-based approach for short text clustering. Proceedings of the 20th ACM International Conference on Knowledge Discovery and Data Mining (SIGKDD); 2014 Aug 24–27; New York, USA. USA: ACM; 2014. p. 233–242.
